# Identification of diarrheagenic *Escherichia coli *isolated from infants and children in Dar es Salaam, Tanzania

**DOI:** 10.1186/1471-2334-7-92

**Published:** 2007-08-09

**Authors:** Sabrina J Moyo, Samwel Y Maselle, Mecky I Matee, Nina Langeland, Haima Mylvaganam

**Affiliations:** 1 Department of Microbiology and Immunology, School of Medicine, Muhimbili University College of Health Sciences, Dar es Salaam, Tanzania; 2 Department of Microbiology and Immunology, Haukeland University Hospital, Bergen, Norway; 3 Department of Medicine, Haukeland University Hospital, Bergen, Norway; 4 Institute of Medicine, University of Bergen, Bergen, Norway

## Abstract

**Background:**

Relatively few studies have been done in Tanzania to detect and classify diarrheagenic *Escherichia coli *(DEC) strains among children with diarrhea. This study aimed at investigating DEC among children in Dar es Salaam aged less than five years hospitalized due to acute/persistent diarrhea.

**Methods:**

DEC were isolated from stool samples collected from two hundred and eighty children with acute/persistent diarrhea at Muhimbili National Hospital and Ilala and Mwananyamala Municipal Hospitals in Dar es Salaam. A multiplex PCR system method was used to detect a species specific gene for *E.coli *and ten different virulence genes for detection of five pathogroups of DEC namely enteroaggregative- (EAEC), enteropathogenic- (EPEC), enterotoxigenic- (ETEC), enteroinvasive- (EIEC) and enterohemorghagic- *Escherichia coli *(EHEC).

**Results:**

Sixty-four patients (22.9%) harbored DEC. Forty-one of them (14.6%) were categorized as EAEC. Most of the EAEC (82.9%) were classified as typical EAEC possessing the *aggR *gene, and 92.6% carried the *aat *gene. Isolates from thirteen patients were EPEC (4.6%) and most of these (92.3%) were typical EPEC with both *eae *and *bfpA *genes. Ten isolates were identified as ETEC (3.6%) with only the heat stable toxin; either *st1a *or *st1b *but not both. Age wise, EAEC and EPEC were significantly more prevalent among the age group 0–6 months (p < 0.05). Genes for EHEC (*stx*_1 _and *stx*_2_) and EIEC *(ial*) were not detected in this study group.

**Conclusion:**

The results show a high proportion of DEC among Tanzanian children with diarrhea, with typical EAEC and typical EPEC predominating. The use of primers for both variants of ST1 (st1a and st1b) increased the sensitivity for detection of ETEC strains.

## Background

Diarrhea is one of the leading causes of morbidity and mortality among children under five years in the developing world [1]. During the period from 1950 to 1970s it was estimated that 4.6 million children died annually from diarrhea in developing world [2,3]. Mortality due to diarrhea declined to approximately 3.3 million annually in the 1980s [1,3]. Currently diarrhea has been reported to account for 1.6–2.5 million deaths annually [3,4]. Despite the decline in mortality, diarrhea still remains one of the principal causes of morbidity in the developing world, with each child experiencing an average of three episodes of diarrhea per year[4]. In these countries, diarrheal diseases are the second most common illness of children after acute respiratory illness[5]. The causes of diarrhea include a wide range of viruses, bacteria, and parasites[6]. Among the bacterial causes diarrheagenic *Escherichia coli *(DEC) is the most important etiologic agent of childhood diarrhea and represents a major public health problem in developing countries[7]. Identification of DEC strains requires that these organisms be differentiated from non-pathogenic members that constitute normal intestinal flora. Molecular identification and classification of DEC is based on the presence of different chromosomal or plasmid-encoded virulence genes, which are absent in the commensal *E.coli*. Further features that supplement such categorization include the effects produced by the proteins encoded by these virulence genes; the pattern of their interaction with intestinal epithelial cells and tissue culture monolayers [7]. DEC strains can be divided into six main categories on the basis of distinct molecular, clinical and pathological features[7]: enteroaggregative *E. coli *(EAEC), enterohemorrhagic (Shiga-toxin producing *E. coli *(EHEC/STEC), enteroinvasive *E. coli *(EIEC), enteropathogenic *E. coli *(EPEC), enterotoxigenic *E. coli *(ETEC) and diffusely adherent *E.coli *(DAEC). Thus, identification of different types of DEC includes biochemical reactions, serotyping, phenotypic assays based on virulence characteristics and molecular detection methods[7]. Among these, detection of specific virulence genes by polymerase chain reaction (PCR) is frequently used because this method gives rapid, reliable results with a high sensitivity and a high specificity[8,9]. The epidemiological significance of each DEC category in childhood diarrhea varies with geographical area. Few studies have been done in Tanzania to detect DEC strains among children with diarrhea [5,10]. One recent study has been done in Ifakara, Tanzania, which is 500 km from Dar es Salaam [5]. In Dar es Salaam only one study was conducted ten years ago to detect DEC using the DNA probe methods [10]. It is known that DNA probes tend to have a lower sensitivity and specificity than the PCR-based method [11]. Furthermore, the study included children with chronic diarrhea only, even though acute diarrhea is more predominant among children in Tanzania [5]. The aim of this study is to report epidemiological data of the different categories of DEC, 10 years after the previous one, in children with both acute and persistent diarrhea, aged less than five years, in Dar es Salaam, Tanzania and using a more reliable PCR-based method.

## Methods

### Study design, population and settings

This was a prospective cross-sectional study that was conducted in Dar es Salaam, Tanzania between December 2005 and February 2006. Participants were children ≤ 5 years, who during the study period, were admitted due to diarrhea at Muhimbili National Referral Hospital (MNH) and Amana, Mwananyamala, and Temeke Municipal Hospitals in Dar es Salaam, Tanzania. Enrolment was subject to obtaining an informed verbal consent from parent or guardian who accompanied the child.

### Interviews

A standard structured questionnaire was used to obtain information of the children from the parents/guardians. Information that was sought included age, sex, duration and description of the stool, as watery, mucoid, or bloody. Diarrhea was defined, according WHO guidelines [12], as the occurrence of three or more, loose, liquid, or watery stools within 24 hours. The guidelines stipulate three forms of diarrhea namely: i) acute watery diarrhea defined as diarrhea that begun acutely and lasted less than or equal to 13 days, ii) dysentery defined as mucoid bloody stool associated with anorexia, abdominal cramps, and tenesmus and iii) persistent diarrhea defined as diarrhea with a duration of 14 or more days. Information was also sought regarding the use of antibiotics prior to hospitalization.

### Weight measurements

Infants under two years of age were weighed using a 25 kg Salter hanging scales (CMS Weighing equipment, High Holborn, London, United Kingdom). Children over two years were weighed on scales calibrated before each session. Weight of children was recorded to the nearest kilogram.

### Determination of nutritional status

Weight-for age Z-scores were calculated using EPI Info (USD, Inc., Stone Mountain, GA). According to WHO criteria Children were considered to be undernourished if the Z-scores were less than -2SD[13].

### Collection and transportation of stool

Stool specimens from enrolled children were collected using wide mouthed sterile plastic containers and transported immediately to the microbiology laboratory for analysis within two hours of collection.

### Bacteriological procedures

Samples were plated on MacConckey Agar (MCA), Xylose lysine Deoxycholate (XLD), Salmonella Shigella Agar (SSA), Thiosulphate Citrate Bile-salt Sucrose Agar (TCBS) (Remel Microbiology Products, Lenexa, KS) and incubated aerobically at 37°C overnight for the isolation of *E.coli*, *V.cholerae*, *Shigella *species, and *Salmonella *species. Red colonies on MCA which were Gram-negative rods and oxidase negative were provisionally identified as *E.coli *by a positive indole test. Since there is a possibility of picking up a commensal non-diarrheagenic colony of *E.coli*, three to five colonies of the bacterium from the primary streak of each fecal sample were sub-cultured on a nutrient agar slant for later analysis for DEC.

### Detection of virulence factors of diarrheagenic *E.coli*

All species provisionally identified as *E.coli *were further examined for the presence of an *E.coli *specific *uidA *gene and for genes coding for virulence factors of ETEC, EPEC, EHEC, EIEC and EAEC using a multiplex PCR system method as described previously [9,14] but with an annealing temperature of 55°C. A sweep of growth on nutrient agar slants was used for the PCR.

To extract DNA a sweep of growth on a nutrient agar slant were boiled in 500 μL of sterile distilled water for 10 minutes. Then centrifugation was done at 13000 rpm for 5 minutes to pellet the cell debris. 1.0 μL of the supernatant was used as template for the PCR amplification. Positive and negative controls were used with each PCR set up. The positive controls were either reference strains (*uidA *gene; *E.coli *ATCC 43889, *eae *and *bfpA *genes (EPEC); ATCC 43887,*elt *and *st1a *genes, (ETEC); ATCC 35401, and *stx*_1 _and *stx*_2 _(EHEC); ATCC 43889) or strains known to posses the target genes *st1b *gene (ETEC), *ial *gene (EIEC) and *aat *and *aggR *genes (EAEC), verified by sequencing of the amplified genes. Sterile distilled water was used as a negative control.

For the detection of ETEC, EPEC, EIEC, and EHEC initially three assays, n1, n2 and n3 were used as published earlier with some modifications [9]. Assay n1 utilized primer pairs for genes coding for heat stable toxin ST (*stIa*) and heat labile toxin LT (*elt*) of ETEC, and (*uid*A) for the *E.coli *β-glucuronidase. Assay n2 detected the presence of the *eae *(structural gene for intimin found in EPEC) and *bfp*A (structural gene for the bundle-forming pilus) of EPEC, and assay n3 screened for *stx*_1 _and *stx*_2 _of shiga toxin producing EHEC and *ial *(invasion-associated locus of the invasion plasmid) found in EIEC/*Shigella*.

Primers of a variant gene *st1b *coding for the heat stable toxin of ETEC were subsequently included in a separate tube in assay n4 together with *uidA *gene. Two plasmid genes of EAEC namely *aat *and *aggR *were detected simultaneously in assay n5. Table 1 shows the primer nucleotide sequence used and the predicted length of the amplicon for each primer pair.

**Table 1:** Primer sequence, primer concentration and predicted length of PCR products for multiplex PCR used to identify ETEC, EPEC, EHEC, EIEC and EAEC

Assay	Target gene	Oligonucleotide sequence (5' to 3')	Ampl Size (bp)	Reff
	*elt*	F. CTCTATGTGCACACGGAGC	322	12
		R. CCATACTGATTGCCGCAAT		
Assay n1	*St1a*	F.TCTTTCCCCTCTTTTAGTCAGTC	170	11
		R. CCGCACAGGCAGGATTAC		
	*uid*A	F. CCAAAAGCCAGACAGAGT	623	11
		R. GCACAGCACATCAAAGAG		

	*eae*	F. TGATAAGCTGCAGTCGAATCC	229	11
Assay n2		R. CTGAACCAGATCGTAACGGC		
	*bfp*A	F. CACCGTTACCGCAGGTGTGA	450	11
		R. GTTGCCGCTTCAGCAGGAGT		

	*stx*_1_	F. GAAGAGTCCGTGGGATTACG	130	11
		R. AGCGATGCAGCTATTAATAA		
Assay n3	*Stx*_2_	F.GGGTACTGTGTGCCTGTTACTGG	510	11
		R. GCTCTGGATGCATCTCTGGT		
	*ial*	F.CTGGTAGGTATGGTGAGG	320	11
		R. CCAGGCCAACAATTATTTCC		

	*St1b*	F.ATTTTTCTTTCTGTATTGTCTT	190	15
Assay n4		R.CACCCGGTACAAGCAGGATT		
	*uid*A	F. CCAAAAGCCAGACAGAGT	623	11
		R. GCACAGCACATCAAAGAG		

	*aggR*	F. CTAATTGTACAATCGATGTA	457	12
Assay n5		R. AGAGTCCATCTCTTTGATAAG		
	*Aat*	F. CTGGCGAAAGACTGTATCAT	629	12
		R. CAATGTATAGAAATCCGCTGTT		

**F**. forward primers, **R**. reverse primers.

PCR was carried out with 1.0 μL of the template added to 24 μL mix containing 10 picomoles of each primer and HotStarTaq Master Mix (QIAGEN, Norway, containing HotStarTaq DNA polymerase, PCR Buffer (with 3 mM MgCl_2_), and 400 μM of each dNTP). Cycling conditions were initial Hotstar Taq DNA polymerase enzyme activation 95°C for 15 minutes, followed by 30 cycles of denaturation at 94°C for 1 minute, annealing at 55°C for 1 minute, extension at 72°C for 1 minute and final extension at 72°C for 10 minutes.

The amplicons were checked by gel electrophoresis and their size determined by using 100 bp DNA molecular weight marker XIV (Roche Diagnostics). The gel electrophoresis was set at 125 V and run for 1 hour and 15 minutes. The products were visualized using a UV transilluminator.

### Interpretation of results

Only the presence of the amplification product with correct sized was interpreted as a positive test (table 1). The presence of *uid*A gene was regarded as confirmation of *E.coli *identification and served as an internal PCR control.

### Ethical considerations

Ethical and research clearance was obtained from the Higher Degree Research and Publication Committee of the Muhimbili University College of Health Sciences in Dar es Salaam, Tanzania. Permission to conduct the study was sought from the respective hospital authorities and the Dar es Salaam City Medical Officer. Informed verbal consent was obtained from parents/guardians of the child before enrolment into the study. The following information was given to ensure that the parents/guardians have the information needed to make an informed choice: a complete description of the aims of the study, and the potential benefits and risks, if any. Study personnel provided any other requested additional information. Children were treated by their attending clinicians according to integrated management of childhood infections (IMCI) guidelines [12] where rehydration therapy is the mainstay of treatment. Antibiotics were given to suspected cases of cholera, dysentery and persistent diarrhea, in accordance with the antimicrobial sensitivity results of bacterial pathogens, namely, *Salmonella*, *Shigella *and *Vibrio cholerae*. All patients' information and test results were confidentially kept.

### Data analysis

The Statistical Package for the Social Sciences (SPSS 10.0) was used for statistical analysis. Assuming the data follows a normal distribution, comparison of proportions and statistical significance were tested by using the Chi-square test. A p value less than 0.05 was considered statistically significant.

## Results

During the study period from December 2005 to February 2006 a total of 280 children with diarrhea were included in this study. These children were aged between 0 and 60 months, with most of them (90.4%) being below 24 months (Mean 12.9 months). DEC were detected in 64 patients (22.9%).

Of the DEC, EAEC were the most prevalent, accounting for 14.6% of all cases of diarrhea. Of a total of 41 isolates of EAEC, 34 (82.9% of EAEC) were classified as typical EAEC, harboring the *aggR *gene, of which 31 isolates had *aat *gene in addition. A total of 38 (92.7%) were *aat *gene positive (Table 2). Table 3 shows the association between different categories of DEC with social demographic data, nutritional status, breast-feeding behavior and type of diarrhea. Thirty-five (85.4%) of the 41 children with EAEC presented with acute watery diarrhea, while five (12.2%) and one (2.4%) presented with persistent diarrhea and dysentery respectively. There were a total of 13 EPEC (4.6% of diarrhea cases), and 12 (92.3%) were typical EPEC with both *eae *and *bfpA *genes and one strain was atypical with only the *eae *gene. Among children harboring EPEC, ten (76.9%) had acute watery and three (23.1%) had persistent diarrhea. ETEC were found in 10 patients (3.6%of all the diarrhea cases). None of the ETEC strains had labile toxin. All ETEC strains had either stable toxins *stIa *or *st1b *but not both. Six (60.0%), two (20.0%) and two (20.0%) of the children with ETEC had acute watery diarrhea, persistent diarrhea and dysentery, respectively. Nine patients had mixed infections; three of them harbored ETEC and EAEC, two harbored EPEC and EAEC three patients harbored ETEC and *Shigella *spp, while one harbored EAEC and *Shigella *spp, (Table 2).

**Table 2:** Distribution of virulence genes among the isolated DEC

DEC (type and genes) n = 64	Number (%)	% of all children (n = 280)	% within DEC type n = 64
EAEC	41	14.6	64.1
-*aggR*	34(82.9)		
-*aat*	38(92.7)		
-*aggR *+ *aat*	31(75.6)		
EPEC	13	4.6	20.3
-*eae*	1(7.7)		
-*eae *+ *bfpA*	12(92.3)		
ETEC	10	3.6	15.6
-*elt*	0 (0.0)		
-*St1a*	5(50.0)		
-*St1b*	5(50.0)		
EIEC	0	0.0	0.0
-*ial*			
EHEC	0	0.0	0.0
-*stx*_1 _or *stx*_2_			
Mixed infection	9	3.2	14
ETEC + EAEC	3		
EPEC + EAEC	2		
ETEC + *Shigell*a spp	3		
EAEC+ *Shigell*a spp	1		

**Table 3:** Association between different categories of DEC with social demographic data, nutritional status, breast feeding behavior and type of diarrhea (percentage in brackets)

	EAEC (n = 41)	EPEC (n = 13)	ETEC (n = 10)	Other^a ^(n = 216)	Total (n = 280)
*Age in months*					
0–6	14(27.5)^b^	7(13.7)^b^	2(3.9)	28(54.9)	51
7–12	21(15.6)	4(3.0)	5(3.7)	105(77.8)	135
13–24	4(6.0)	2(3.0)	1(1.5)	60(89.6)	67
25–60	2(7.4)	0(0.0)	2(7.4)	24(82.5)	27
*Sex*					
Boys	25(14.5)	11(6.4)	4(2.3)	132(76.7)	172
Girls	16(14.8)	2(1.9)	6(5.6)	84(77.8)	108
*Diarrhea type*					
Acute	35(14.9)	10(4.3)	6(2.6)	184(78.3)	235
Persistent	5(12.2)	3(11.1)	2(7.4)	17(63.0)	27
Dysentery	1(2.4)	0(0.0)	2(11.1)^c^	15(83.3)	18
*Exclusive BF*^d^					
Yes	1(16.7)	0(0.0)	0(0.0)	5(83.3)	6
No	13(28.9)	7(15.6)	2(4.4)	23(51.1)	45
*Nutritional status*					
Normal	25(12.4)	13(6.5)^b^	4(5.1)	159(79.1)	201
Malnourished	16(20.3)	0(0.0)	6(3.0)	57(72.2)	79

^a ^No DEC found; ^b^P value < 0.05; ^c^These two patients with dysentery and ETEC strains were co-infected with *Shigella *species; ^d"^BF" = breast feeding, only calculated for the age group 0–6 months

There were no EIEC or EHEC strains detected in this study group. EAEC and EPEC were significantly more prevalent among the age group 0–6 month's (p < 0.05). Of these children below six months of age, 88.2% were not on exclusive breast-feeding. Seventy nine children were malnourished and of these, 20.3% and 3.0% harbored EAEC and ETEC strains of DEC respectively.

## Discussion

Our results show a high proportion of EAEC accounting for 64.1% of DEC. This study was conducted during the dry season of the year and these findings are in agreement with a previous study among children of Ifakara Tanzania in which, EAEC accounted 63% of the DEC during dry season [5] and with other studies in other developing countries [15-18]. Collectively, these studies seem to suggest the predominance of EAEC among DEC in causing diarrhea in children in developing countries. It is worthy noting that the proportion EAEC among children aged less than six months is significantly higher than in older children (p < 0.05), which is in agreement with the finding of Gonzalez et al [19]. However, we noted age differences when our results are compared with findings of other countries, with a higher prevalence of EAEC in infants aged less than six months [15,17]. In these studies most of the children younger than six months were exclusively breast fed. Correspondingly the fact that most children in the present study (88.2%) were not exclusively breast fed may explain the discrepancies.

In the present study, the plasmid-borne genes *aggR *and *aat *were used to detect EAEC. The pathogenic mechanisms of EAEC infection are only partially understood. The varying presence of the different virulence factors indicates heterogeneity of the EAEC isolates [15]. It has been hypothesized that the combination of these genes increases strain virulence. The *aggR *gene is a transcriptional activator gene required for the expression of aggregative adherence fimbria I gene (*aagA*) [20,21] and was subsequently also shown to be required for aggregative adherence fimbria II gene (*aafA*) expression [22]. More recently, *aggR *has been shown to be required for the expression of the antiaggregation protein (dispersin) gene *aap *(previously called *aspU*) [16] and an anti-aggregation protein transporter gene *aatA *(*previously called CVD432 or *AA probe) [23] genes. Therefore, it is likely that the *aggR *gene serves as a marker for truly virulent EAEC strains, which causes expression of a package of virulence genes. Nataro has recently suggested the term "typical EAEC" to refer to strains expressing the *aggR *regulon [24]. Our data shows a high prevalence of typical EAEC than atypical among diarrheic children of Dar es Salaam which is in agreement with the findings of Sarantuya *et al *[16]. Other epidemiological studies have suggested greater pathogenicity of *aat*-positive strains than of *aat*-negative strains [25].

In the present study EPEC was the second most common DEC isolated and most of these were typical EPEC with both *eae *and *bfpA *genes. Typical EPEC is well recognized as a cause of gastroenteritis in infants[7]. In our study we detected only one strain of atypical EPEC in a child with diarrhea in contrast with the finding of other study in Ifakara Tanzania who found a much higher percentage of atypical EPEC [5]. The discrepancy can be, at least partly, attributed to geographical differences. The role of atypical EPEC in childhood diarrhea still remains controversial [26,27].

ETEC in the present study accounted for 15.6% of the DEC, which is high compared with the finding of Cegielski et al (11.9%,1996) in Dar es Salaam Tanzania [10]. However, our findings were lower than those reported from Ifakara, Tanzania (20% and 51.6% during dry and rainy seasons, respectively [5]. These differences could possibly be related with seasonality as well as methodological issues. It is known that ETEC strains cause diarrhea through the action of the enterotoxins LT (labile toxin) and ST (stable toxin) and that there are two distinct classes of STs that differ in structure and mechanism of action STI and STII [25]. There are two variants of STI designated *st1a *(STp porcine) and *st1b *(STh human), both variants can be found in human ETEC strains [7,28]. Unlike most studies where ETEC strains were detected by looking for only one variant of ST [5,29], in our study, ETEC was detected by looking for both variants of STI. Detection of both variants of STI increases sensitivity detecting ETEC. This argument is supported by our findings showing that detection of ETEC would have had a decreased sensitivity of 50% if only either of the gene variants of ST1 had been screened for. Furthermore, it was noted that all the ETEC strains harbored only the heat-stable (ST) enterotoxin genes but not the heat-labile enterotoxin (LT) genes. Our findings showing a higher prevalence of ST producing than LT producing ETEC are in keeping with the observations of Vargas et al [5] in Ifakara, Tanzania and [5] and of other studies conducted elsewhere [29,30]. Collectively, these studies seem to suggest a greater association between ST-producing strains and diarrhea than LT producing ETEC.

Finally, we did not find EHEC and EIEC strains, which is in agreement with the previous study in Tanzania salaam [5,10], indicating their limited role in childhood diarrhea in Tanzania.

## Conclusion

Our results show a high proportion of DEC, where typical EAEC and typical EPEC predominate among Tanzanian children with diarrhea. We also show that the use of primers (*st1a *and *st1b*) for both variants of ST1 increases the sensitivity for detection of ETEC strains.

## Competing interests

The author(s) declare that they have no competing interests.

## Authors' contributions

SJM was the principal investigator, who conceived and designed study and was responsible for collection of specimens and clinical information as well as data analysis. Laboratory investigations were performed by SJM, the molecular biological part under the guidance of HM. SYM, MIM, NL and HM assisted in the development of the research proposal, data analysis and preparation of the manuscript.

## Pre-publication history

The pre-publication history for this paper can be accessed here:


